# Association of Carboxyhemoglobin With Severity and Outcomes in Hypothermic Patients: A Retrospective Cohort Study

**DOI:** 10.7759/cureus.97962

**Published:** 2025-11-27

**Authors:** Yuya Miyoshi, Tetsuya Yumoto, Takashi Hongo, Takafumi Obara, Tsuyoshi Nojima, Hiromichi Naito, Atsunori Nakao

**Affiliations:** 1 Department of Emergency, Critical Care, and Disaster Medicine, Faculty of Medicine, Dentistry, and Pharmaceutical Sciences, Okayama University, Okayama, JPN

**Keywords:** carbon monoxide, carboxyhemoglobin, heme oxygenase, hypothermia, sepsis

## Abstract

Introduction

Carboxyhemoglobin (COHb), an endogenous marker of carbon monoxide production mediated by heme oxygenase-1, may reflect physiological stress responses in critically ill patients. However, its clinical relevance in accidental hypothermia remains unclear.

Methods

We conducted a single-center retrospective cohort study of adult patients admitted to the emergency ICU with accidental hypothermia between January 1, 2019, and March 31, 2025. Patients were categorized into low- and high-COHb groups based on median COHb levels upon emergency department arrival. Associations between COHb levels, disease severity (Acute Physiology and Chronic Health Evaluation II (APACHE II) and Sequential Organ Failure Assessment (SOFA) scores), and 28-day mortality were analyzed using regression models adjusted for clinical confounders.

Results

Among the 88 patients, who had a median admission temperature of 28.7°C, 45 were classified into the low-COHb group and 43 into the high-COHb group, based on a median COHb level of 0.3%. Lower COHb levels on admission were significantly associated with higher APACHE II scores (β = −4.20; 95% CI, −7.56 to −0.85), but not with SOFA scores. Admission and minimum COHb levels were not associated with 28-day mortality. However, higher maximum COHb levels within the first 24 hours were independently associated with lower 28-day mortality (adjusted OR, 0.17; 95% CI, 0.023 to 0.93).

Conclusions

Lower COHb levels were associated with greater disease severity, and higher maximum COHb levels were associated with lower 28-day mortality. COHb may reflect systemic stress in accidental hypothermia, but its prognostic value appears limited.

## Introduction

Accidental hypothermia is a life-threatening condition that significantly increases morbidity and mortality through multi-organ dysfunction [1]. Accidental hypothermia is defined as a core body temperature below 35°C. It disrupts multiple physiological systems, leading to cardiovascular instability, altered consciousness, renal impairment, and coagulopathy [2]. While it often arises in severe conditions such as sepsis or trauma, hypothermia itself constitutes a critical clinical state, necessitating prompt recognition and intervention to improve outcomes.

Carbon monoxide (CO) modulates multiple intracellular signaling pathways, including those mediated by soluble guanylate cyclase [3,4]. Endogenously, CO is produced via the enzymatic activity of heme oxygenase (HO), primarily its isoforms HO-1 and HO-2. HO-1, an inducible enzyme, is upregulated not only by its substrate heme but also by oxidative and other cellular stressors. Given its physiological role, CO has been investigated as a biomarker through carboxyhemoglobin (COHb) levels in critically ill patients, including those experiencing cardiac arrest, where several studies have reported that lower COHb levels were associated with worse outcomes [5-9].

Although COHb has been studied in various critical care settings, its relevance in the context of accidental hypothermia has not been well established. Given that accidental hypothermia induces oxidative stress, which may modulate endogenous CO production via the heme oxygenase pathway [10], we explored whether COHb levels reflect illness severity or prognosis in this population. 

We hypothesized that lower COHb levels on admission would be associated with greater disease severity and poorer short-term outcomes in patients with accidental hypothermia. To test this hypothesis, we examined COHb measured at emergency department (ED) arrival and the prespecified maximum COHb level within the first 24 hours of admission. COHb was analyzed as a continuous variable in all inferential analyses, while median-based grouping was used only for descriptive comparisons. The primary outcome was 28-day mortality, and secondary outcomes were early disease severity assessed by Acute Physiology and Chronic Health Evaluation II (APACHE II) and Sequential Organ Failure Assessment (SOFA) scores.

## Materials and methods

Study design, population, and ethics

This single-center retrospective cohort study was conducted at Okayama University Hospital, a tertiary care center that exclusively receives emergency patients transported by emergency vehicles.

We included adult patients (aged ≥18 years) who were diagnosed with accidental hypothermia in the ED of our hospital between January 1, 2019, and March 31, 2025. Patients were identified using the Japanese Diagnosis Procedure Combination inpatient database and the International Classification of Diseases, Tenth Revision (ICD-10) code T68, which denotes hypothermia. All cases identified via ICD-10 were subsequently validated by two independent investigators through manual review of ED and emergency medicine services records to confirm accidental (primary) hypothermia and exclude coding misclassification. Exclusion criteria were as follows: missing core body temperature (measured via esophageal, bladder, or rectal routes) or COHb values; a recorded core body temperature ≥35.0°C in the ED; out-of-hospital cardiac arrest; and lack of admission to the emergency intensive care unit (ICU). Out-of-hospital cardiac arrest was identified and excluded based on emergency medical services records and ED documentation.

This study was approved by the Okayama University Hospital Ethics Committee (approval number K2409-019) and was conducted in accordance with the principles outlined in the Declaration of Helsinki. Given the retrospective nature of the study and the use of anonymized data, the ethics committee waived the requirement for written informed consent.

Data collection

Data collected included basic patient characteristics such as age, sex, smoking status, Charlson Comorbidity Index, and the etiology of hypothermia (including trauma/environmental, metabolic/endocrine, drug/alcohol-related, sepsis, stroke, frailty, and other medical conditions) [11]. The etiology of hypothermia was determined by the investigator's chart review, assigning each patient to the most likely primary cause based on clinical and diagnostic information. Additional variables included prehospital oxygen administration, vital signs upon ED arrival, and blood gas analysis on arrival. All patients received active rewarming. Core temperature was continuously or periodically monitored according to standard ICU protocols, and the time to achieve normothermia (≥35.0°C) was recorded.

Regarding ICU interventions, disease severity, and outcomes, the following data were collected: use of mechanical ventilation, renal replacement therapy, extracorporeal membrane oxygenation, and serial COHb levels within the first 24 hours of ICU admission. Disease severity was assessed using the APACHE II and SOFA scores calculated from the worst values during the first 24 hours after ICU admission, representing early 24-hour severity. In-hospital and 28-day mortality were also documented.

Blood gas analysis and COHb level measurement

Blood gas analyses were performed immediately upon hospital arrival using heparinized blood samples, either venous or arterial, depending on the attending physician’s preference. COHb levels, along with oxygen and carbon dioxide partial pressures (PO₂, PCO₂), pH, and lactate levels, were measured using the ABL800 FLEX analyzer (Radiometer Medical ApS, Brønshøj-Husum, Denmark). To ensure measurement accuracy, the analyzer undergoes automatic zero calibration of its optical system against a colorless calibration fluid at least every 8 hours. No samples were stored or refrigerated. In all cases, the time from blood draw to analyzer measurement was <3 minutes. The timing and frequency of COHb measurements in the emergency ICU were determined at the discretion of the attending physician. All samples obtained in the emergency ICU were arterial.

Grouping and outcomes

Patients were divided into two groups: the low-COHb group and the high-COHb group, based on the median COHb level upon hospital arrival. The primary outcome was 28-day mortality. Secondary outcomes included disease severity and organ dysfunction, assessed using APACHE II and SOFA scores on the day of ICU admission.

Data analysis

Continuous variables are presented as medians with interquartile ranges (IQRs), and categorical variables are expressed as counts and percentages. The chi-square test was used to compare categorical variables, while the Mann-Whitney U test was used for continuous variables. 

The correlation between COHb levels and disease severity, assessed by APACHE II and SOFA scores, was evaluated using Spearman’s correlation coefficient. Multiple linear regression analyses were performed to assess the adjusted association between COHb levels on admission and APACHE II and SOFA scores. Results are reported as β coefficients with corresponding 95% confidence intervals (CIs). Multiple logistic regression models were used to estimate adjusted odds ratios (ORs) and 95% CIs for 28-day mortality. These models were adjusted for potential confounders, including age, sex, prehospital oxygen dose, core temperature on admission, sepsis as the underlying cause of hypothermia, and lactate level on admission, based on previous studies [12-15]. Given its potential to affect COHb levels, prehospital oxygen administration was included as a covariate in the adjusted models. Model calibration was evaluated using Hosmer-Lemeshow tests, and discrimination using AUC (area under the curve) statistics. As a sensitivity analysis, we additionally included a binary variable indicating any prehospital oxygen administration to confirm the robustness of the results. Furthermore, we performed a sensitivity analysis adjusting for the sampling site (arterial vs venous) to address potential bias in admission COHb levels. Finally, to evaluate the possibility of informative-measurement bias in the maximum and minimum COHb values within the first 24 hours of ICU admission, we conducted an additional sensitivity analysis adjusting for the number of COHb measurements per patient.

A p-value <0.05 was considered statistically significant. Statistical analysis was performed using STATA Now/SE 19.5 (StataCorp, Lakeway, TX) and ﻿Prism 10.0.3 (GraphPad, San Diego, CA).

## Results

**Figure 1:**
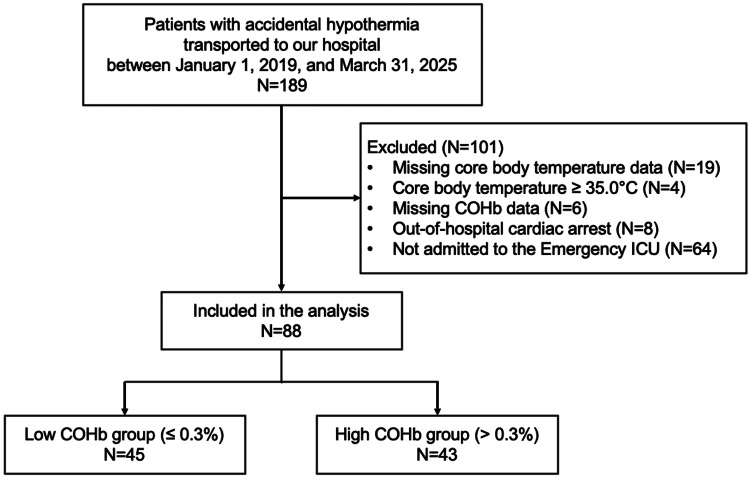
Patient flow chart. COHb: carboxyhemoglobin; ICU: intensive care unit.

During the six-year and three-month study period, a total of 189 patients with accidental hypothermia were identified, of whom 88 met the inclusion criteria and were analyzed. The study flowchart is shown in Figure 1. 

Patient characteristics

Demographic and clinical characteristics of the study population are summarized in Table 1. The median age was 79 years (IQR, 65-87), and 39 patients (44.3%) were women. The median core body temperature on admission was 28.7°C (IQR, 27.0-31.6). The most common cause of hypothermia was trauma or environmental exposure (n = 27, 30.7%), followed by sepsis (n = 23, 26.1%) and frailty (n = 17, 19.3%).

**Table 1:** Patient characteristics of the study population. Vital signs and laboratory values were obtained upon arrival at the emergency department. The Mann-Whitney U test was used for U-values, and the chi-square test was used for χ² values. ^a^PaO₂ data were missing for 12 patients in the low-COHb group and six patients in the high-COHb group. COHb: carboxyhemoglobin; ED: emergency department; IQR: interquartile range; GCS: Glasgow Coma Scale.

Variables	All N = 88	Low-COHb group (<0.4%) N = 45	High-COHb group (≥0.4%) N = 43	Test statistic values	p-Value
						Age, median (IQR), years	79 (65-87)	80 (70-86)	78 (63-88)	U = 927.5	0.738
						Sex, n (%)				χ^2^ = 1.10	0.206
Men	49 (55.7)	28 (62.2)	21 (48.8)		
Women	39 (44.3)	17 (37.8)	22 (51.2)		
Smoking status, n (%)				χ^2^ = 2.19	0.550
Never smoker	45 (51.1)	25 (55.6)	20 (46.5)		
Past smoker	13 (14.8)	7 (15.6)	6 (13.9)		
Current smoker	10 (11.4)	3 (6.7)	7 (16.3)		
Unknown	20 (22.7)	10 (22.2)	10 (23.3)		
Charlson Comorbidity Index, median (IQR)	1 (0-2)	1 (0-2)	1 (0-2)	U = 960	0.948
Etiology of hypothermia, n (%)				χ^2^ = 10.19	0.126
Trauma/Environmental	27 (30.7)	13 (28.9)	14 (32.6)		
Metabolic/Endocrine	4 (4.5)	4 (8.9)	0 (0.0)		
Drug/Alcohol-related	4 (4.5)	0 (0.0)	4 (9.3)		
Sepsis	23 (26.1)	12 (26.7)	11 (25.6)		
Stroke	2 (2.3)	2 (4.4)	0 (0.0)		
Frailty	17 (19.3)	9 (20.0)	8 (18.6)		
Other medical conditions	11 (12.5)	5 (11.1)	6 (14.0)		
Prehospital oxygen administration, median (IQR), L/min	10 (0-10)	10 (5-10)	6 (0-10)	U = 727	0.032
Core body temperature, median (IQR), °C	28.7 (27.0-31.6)	28.1 (27.0-30.7)	29.7 (27.3-32.1)	U = 758.5	0.058
GCS, median (IQR)	9 (6-11.5)	8 (6-11)	10 (6-13)	U = 754.5	0.074
Respiratory rate, median (IQR), /min	19 (15.5-23)	18 (15-21)	20 (16-25)	U = 741	0.058
Heart rate, median (IQR), /min	61 (45-89)	60 (45-85)	62 (45-92)	U = 888	0.507
Systolic blood pressure, median (IQR), mmHg	113 (82-136)	101 (76-136)	115 (90-135)	U = 809	0.186
Arterial blood gas sample, n (%)	70 (79.5)	33 (73.3)	37 (86.0)	χ^2^ = 1.47	0.139
Blood gas analysis on ED arrival					
pH, median (IQR)	7.27 (7.20-7.33)	7.21 (7.01-7.30)	7.31 (7.26-7.36)	U = 434	<0.001
PCO_2_, median (IQR), mmHg	41.2 (33.1-52.0)	39.2 (30.5-45.3)	43.0 (33.6-56.9)	U = 791.5	0.142
PaO_2_^a^, median (IQR), mmHg	145.7 (105.9-307.9)	210.1 (119-392.1)	134 (90.8-256)	U = 451.5	0.061
Base excess, median (IQR), mmol/L	-6.5 (-11.7 to -2.4)	-10.9 (-18.3 to -4.9)	-3.5 (-8.0 to -1.4)	U = 446	<0.001
Lactate, median (IQR), mmol/L	3.1 (1.8-6.8)	4.4 (2.7-8.0)	2.4 (1.5-5.9)	U = 684	0.018
COHb, median (IQR), %	0.3 (0.1-0.7)	0.1 (0.0-0.2)	0.7 (0.5-1.1)	U = 0	<0.001
Glucose, median (IQR), mg/dL	146 (100-217)	141 (93-223)	149 (103-212)	U = 927.5	0.738
Hemoglobin, median (IQR), g/dL	11.8 (10.0-13.7)	12.2 (9.8-13.7)	11.5 (10.4-13.2)	U = 899.5	0.57
Total bilirubin, median (IQR), mg/dL	0.62 (0.39-0.92)	0.64 (0.37-0.90)	0.59 (0.39-0.92)	U = 922	0.704

The median COHb level for the overall cohort was 0.3% (IQR, 0.1-0.7). Accordingly, patients were divided into a low-COHb group (≤0.3%, n = 45) and a high-COHb group (>0.3%, n = 43). Core body temperature tended to be lower in the low-COHb group, although the difference was not statistically significant. Baseline characteristics, including age, sex, Charlson Comorbidity Index, and etiology of hypothermia, did not differ significantly between the two groups. However, the low-COHb group had significantly lower pH (7.21 vs. 7.31, p < 0.001) and base excess (-10.9 vs. -3.5, p < 0.001), higher lactate levels (4.4 vs. 2.4, p = 0.018), and received a higher dose of prehospital oxygen compared to the high-COHb group (10 L/min vs. 6 L/min, p = 0.032).

ICU interventions, disease severity, and outcomes

Table 2 summarizes ICU interventions, disease severity, and outcomes. The median number of COHb measurements per patient was not different between the groups. Patients with low COHb levels had significantly lower maximum (1.0% vs. 1.2%, p < 0.001) and minimum COHb values (0.0% vs. 0.7%, p < 0.001) during the first 24 hours. Disease severity, as assessed by APACHE II scores (30 vs. 23, p < 0.001) and SOFA scores (7 vs. 5, p = 0.012), was significantly greater in the low-COHb group. Although mortality tended to be higher in the low-COHb group, the differences were not statistically significant (in-hospital: 8/45 (17.8%) vs. 3/43 (7.0%), p = 0.126; 28-day: 12/45 (26.7%) vs. 6/43 (13.9%), p = 0.139).

**Table 2:** ICU interventions, disease severity, and clinical outcomes. The Mann-Whitney U test was used for U-values, and the chi-square test was used for χ² values. ^a^Defined as the time from hospital admission to achieving a core body temperature of 35.0°C. ICU: intensive care unit; COHb: carboxyhemoglobin; ECMO: extracorporeal membrane oxygenation; IQR: interquartile range; APACHE: Acute Physiology and Chronic Health Evaluation; SOFA: Sequential Organ Failure Assessment.

Variables	All N = 88	Low-COHb group (<0.4%) N = 45	High-COHb group (≥0.4%) N = 43	Test statistic values	p-Value
						Organ support					
						Ventilator, n (%)	21 (23.9)	16 (35.6)	5 (11.6)	χ^2^ = 6.93	0.008
Renal replacement therapy, n (%)	7 (7.9)	7 (15.6)	0 (0.0)	χ^2^ = 7.27	0.007
ECMO, n (%)	0 (0.0)	0 (0.0)	0 (0.0)	N/A	N/A
Time to achieve normothermia^a^, median (IQR), min	300 (200-480)	300 (180-480)	300 (200-480)	U = 887	0.746
The number of COHb measurements, median (IQR)	4 (4, 5)	4 (4, 5)	4 (3, 5)	U = 780.5	0.101
Maximum COHb during 24 hours after admission, median (IQR), %	1.1 (0.8-1.3)	1.0 (0.7-1.1)	1.2 (1.1-1.4)	U = 461.5	<0.001
Minimum COHb during 24 hours after admission, median (IQR), %	0.2 (0.0-0.7)	0.0 (0.0-0.2)	0.7 (0.4-1.0)	U = 242.5	<0.001
Average COHb during 24 hours after admission, median (IQR), %	0.69 (0.50-1.02)	0.54 (0.33-0.65)	0.96 (0.73-1.20)	U = 318.5	<0.001
APACHE Ⅱ score, median (IQR)	27 (21-32)	30 (26-36)	23 (18-27)	U = 467	<0.001
SOFA score, median (IQR)	6 (4-9)	7 (5-9)	5 (3-7)	U = 667	0.012
In-hospital mortality, n (%)	11 (12.5)	8 (17.8)	3 (7.0)	χ^2^ = 2.34	0.126
28-day mortality, n (%)	18 (20.5)	12 (26.7)	6 (13.9)	χ^2^ = 2.18	0.139

Correlation between COHb and disease severity

**Figure 2:**
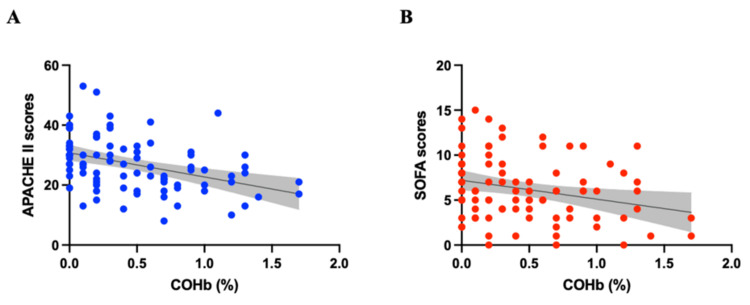
Scatter plot with Spearman’s correlation. (A) APACHE II scores (r = −0.444; 95% CI, −0.602 to −0.252) and (B) SOFA scores (r = −0.264; 95% CI, −0.449 to −0.058). The gray area shows the 95% confidence intervals. APACHE: Acute Physiology and Chronic Health Evaluation; CI: confidence interval; SOFA: Sequential Organ Failure Assessment.

COHb levels showed a moderate negative correlation with APACHE II scores (r = −0.444, 95% CI −0.602 to −0.252, p < 0.001) and a weak negative correlation with SOFA scores (r = −0.264, 95% CI −0.449 to −0.058, p = 0.014) (Figure 2).

Association of COHb with disease severity and outcomes

Multiple linear regression analysis revealed that COHb levels on admission were negatively associated with APACHE II scores (β = −4.20; 95% CI, −7.56 to −0.85); however, no significant association was observed between admission COHb levels and SOFA scores (β = 0.70; 95% CI, −2.08 to 0.73) (Table 3). The regression model was adjusted using clinically relevant covariates and showed acceptable calibration (Hosmer-Lemeshow p = 0.72) and discrimination (AUC = 0.76; 95% CI, 0.62-0.88).

**Table 3:** Multiple linear regression analysis between COHb levels on admission and APACHE II or SOFA scores and multiple logistic regression analysis between COHb levels and 28-day mortality. Adjusted for age, sex, prehospital oxygen dose, admission core temperature, sepsis as the underlying cause of hypothermia, and admission lactate level. COHb: carboxyhemoglobin; APACHE: Acute Physiology and Chronic Health Evaluation; SOFA: Sequential Organ Failure Assessment; CI: confidence interval.

Variables	β coefficient (95% CI)
APACHE II score	-4.20 (-7.56 to -0.85)
SOFA score	0.70 (-2.08 to 0.73)
	Adjusted OR (95% CI)
COHb on admission	0.78 (0.13 to 3.85)
Minimum COHb levels within 24 hours of admission	1.12 (0.19 to 5.60)
Maximum COHb levels within 24 hours of admission	0.17 (0.023 to 0.93)

In the multiple logistic regression analysis for 28-day mortality, neither admission COHb levels (adjusted OR, 0.78; 95% CI, 0.13 to 3.85) nor minimum COHb levels within the first 24 hours (adjusted OR, 1.12; 95% CI, 0.19 to 5.60) were significantly associated with mortality. In contrast, the maximum COHb level during the first 24 hours was significantly associated with lower odds of 28-day mortality (adjusted OR, 0.17; 95% CI, 0.023 to 0.93) (Table 3).

As a sensitivity analysis using a binary variable indicating any prehospital oxygen administration, the results remained consistent. Neither admission COHb levels (adjusted OR, 0.81; 95% CI, 0.14-3.95) nor minimum COHb levels within the first 24 hours (adjusted OR, 1.12; 95% CI, 0.19-5.51) were significantly associated with mortality. In contrast, the maximum COHb level during the first 24 hours was significantly associated with lower odds of 28-day mortality (adjusted OR, 0.18; 95% CI, 0.026-0.95). 

A sensitivity analysis adjusting for sampling site (arterial vs venous) showed no association between admission COHb levels and 28-day mortality (adjusted OR, 0.73; 95% CI, 0.12-3.76). Another sensitivity analysis adjusting for the number of COHb measurements per patient yielded similar results: the maximum COHb level during the first 24 hours remained significantly associated with lower odds of 28-day mortality (adjusted OR, 0.14; 95% CI, 0.022-0.67), whereas the minimum COHb level within the first 24 hours was not significantly associated with mortality (adjusted OR, 0.84; 95% CI, 0.15-3.76).

## Discussion

In this retrospective study of patients with accidental hypothermia, we found that lower COHb levels upon hospital arrival were associated with higher early 24-hour disease severity, as reflected by APACHE II scores, despite being measured at ED arrival. However, neither admission COHb levels nor minimum COHb levels within 24 hours were associated with 28-day mortality. Interestingly, a lower maximum COHb level during the first 24 hours was independently associated with a higher risk of 28-day mortality, although the estimate was imprecise due to the small number of deaths (18/88). This counterintuitive finding should be interpreted cautiously, as it may reflect informative measurement bias, where sicker patients undergo more frequent testing, and the effect of oxygen-related COHb washout. Therefore, this result should be considered hypothesis-generating rather than confirmatory.

One possible explanation for this discrepancy is that admission COHb represents a single static value influenced by prehospital oxygen therapy and sampling timing, potentially underestimating endogenous CO production. In contrast, the maximum COHb level during the early 24 hours may reflect dynamic HO-1 activation in response to stress, with a higher value indicating preserved adaptability and better survival.

COHb levels were significantly associated with APACHE II scores but not with SOFA scores. However, the correlations were weak to moderate in strength, and the scatter plot showed wide variability, indicating that COHb alone is not sufficient to predict disease severity in individual patients. Notably, the direction and strength of the association varied slightly between unadjusted and adjusted analyses, suggesting that the relationship may be model-dependent and influenced by confounding or nonlinearity. This may be due to differences in the scope and timing of the two scoring systems. APACHE II incorporates a broad range of physiological and laboratory variables within the first 24 hours, providing a comprehensive assessment of acute systemic stress. In contrast, SOFA focuses on dysfunction in specific organ systems and is designed to track changes over time [16]. As such, SOFA may be less sensitive to early global derangements captured by COHb levels. For example, thrombocytopenia, a component of SOFA, may develop gradually during the course of hypothermia, potentially reducing the score’s responsiveness in early assessments [17].

Mechanistically, endogenous CO production is mediated by HO-1, a stress-inducible enzyme with cytoprotective, anti-inflammatory, and anti-apoptotic effects. Although HO-1 levels were not directly measured in our study, COHb serves as an indirect marker of its activity. However, any interpretation linking COHb patterns to “inadequate HO-1 response” remains speculative, as neither HO-1 expression nor endogenous CO production was directly measured in this study. The observed association between lower COHb levels and greater disease severity may be partly related to insufficient HO-1 induction or other factors influencing endogenous CO production, although this interpretation remains speculative given that COHb is only an indirect and imperfect marker of HO-1 activity. This differs from observations in post-cardiac arrest studies, where elevated HO-1 levels have been reported in association with worse outcomes, possibly reflecting differences in disease mechanisms, injury burden, or timing rather than a direct causal relationship [18]. While hypothermia has been shown to induce HO-1 expression in experimental ischemia/reperfusion models [19], such findings may not directly apply to clinical hypothermia, as experimental conditions differ substantially in timing and exposure, and the magnitude and timing of this response in critically ill patients remain uncertain. Taken together, these findings suggest that while COHb and HO-1 can reflect systemic stress, their prognostic value may vary depending on disease context, timing, and severity of injury.

Our results suggest that COHb behaves more as a marker of physiological stress response rather than as a prognostic biomarker of mortality. The association between higher early COHb levels and lower mortality likely reflects endogenous HO-1 activation, which exerts cytoprotective and anti-inflammatory effects during critical stress. However, given the modest effect size and limited event count, these findings should be interpreted cautiously and considered hypothesis-generating, because testing frequency was clinician-determined, raising the possibility of informative-measurement bias, and oxygen therapy may differentially accelerate COHb washout across patients. In addition, the small number of deaths leads to imprecise estimates with wide confidence intervals. Thus, the observed associations are exploratory and may reflect sampling artifacts rather than true biological mechanisms.

This study has several limitations. First, this single-center retrospective study had a relatively small sample size, and the limited number of deaths may have led to small-event bias and model instability. As a result, the mortality models should be interpreted cautiously and regarded as exploratory. Second, accidental hypothermia arises from heterogeneous etiologies, and we were unable to fully stratify by underlying condition, which may influence COHb dynamics. Third, although we adjusted for prehospital oxygen administration, we lacked dose × duration information for prehospital and in-hospital oxygen exposure, which may affect COHb elimination and introduce residual confounding. Fourth, heterogeneity in sampling site (arterial vs venous) and pre-analytic variability may have contributed to measurement differences, despite additional sensitivity analyses adjusting for sampling site. Fifth, COHb measurement frequency during the first 24 hours was determined clinically, raising the possibility of informative-measurement bias. Although we performed a sensitivity analysis adjusting for the number of measurements per patient, some bias may remain. Sixth, because only ICU-admitted patients were included, selection bias toward more severe cases is possible, limiting generalizability to milder hypothermia. Finally, COHb is an indirect and imperfect surrogate for HO-1 activity, and mechanistic conclusions remain limited.

These limitations indicate that the association between maximum COHb levels and mortality should be considered hypothesis-generating, and larger multicenter prospective studies are required to validate these findings.

In future studies, predefined COHb metrics and adjustment for cumulative oxygen exposure (dose × time) should be considered. Moreover, incorporating direct or surrogate measures of HO-1 activity, such as exhaled CO or bilirubin by-products, may help clarify the mechanistic link between CO production and clinical outcomes.

## Conclusions

This study demonstrated that lower COHb levels on admission were associated with greater disease severity in patients with accidental hypothermia, while lower maximum levels within 24 hours were linked to higher 28-day mortality. COHb may serve as an indicator of systemic stress in accidental hypothermia through HO-1-mediated endogenous CO production. However, its independent prognostic significance for mortality remains uncertain, and the associations identified in this study should be interpreted as exploratory and hypothesis-generating. Larger prospective studies are needed to validate its clinical relevance and mechanistic implications.

## References

[1] Paal P, Pasquier M, Darocha T (2022). Accidental hypothermia: 2021 Update. Int J Environ Res Public Health.

[2] Bjertnæs LJ, Næsheim TO, Reierth E, Suborov EV, Kirov MY, Lebedinskii KM, Tveita T (2022). Physiological changes in subjects exposed to accidental hypothermia: An update. Front Med (Lausanne).

[3] Motterlini R, Otterbein LE (2010). The therapeutic potential of carbon monoxide. Nat Rev Drug Discov.

[4] Motterlini R, Foresti R (2017). Biological signaling by carbon monoxide and carbon monoxide-releasing molecules. Am J Physiol Cell Physiol.

[5] Melley DD, Finney SJ, Elia A, Lagan AL, Quinlan GJ, Evans TW (2007). Arterial carboxyhemoglobin level and outcome in critically ill patients. Crit Care Med.

[6] Fazekas AS, Wewalka M, Zauner C, Funk GC (2012). Carboxyhemoglobin levels in medical intensive care patients: A retrospective, observational study. Crit Care.

[7] Yanagawa Y (2012). Significance of the carboxyhemoglobin level for out-of-hospital cardiopulmonary arrest. J Emerg Trauma Shock.

[8] Tezel O, Bilge S, Acar YA, Özkan G (2021). Do carboxyhaemoglobin and methaemoglobin levels predict the return of spontaneous circulation and prognosis of cardiac arrest patients?. Int J Clin Pract.

[9] Hongo T, Yumoto T, Naito H (2025). Association of blood carboxyhemoglobin levels with mortality and neurological outcomes in out-of-hospital cardiac arrest. Acute Med Surg.

[10] Alva N, Palomeque J, Carbonell T (2013). Oxidative stress and antioxidant activity in hypothermia and rewarming: Can RONS modulate the beneficial effects of therapeutic hypothermia?. Oxid Med Cell Longev.

[11] Takauji S, Hifumi T, Saijo Y (2021). Accidental hypothermia: Characteristics, outcomes, and prognostic factors-A nationwide observational study in Japan (Hypothermia study 2018 and 2019). Acute Med Surg.

[12] Thomas-Rüddel DO, Hoffmann P, Schwarzkopf D (2021). Fever and hypothermia represent two populations of sepsis patients and are associated with outside temperature. Crit Care.

[13] Carrola A, Romão CC, Vieira HL (2023). Carboxyhemoglobin (COHb): Unavoidable bystander or protective player?. Antioxidants (Basel).

[14] Cao Y, Yao S, Shang J (2022). The combination of lactate level, lactate clearance and APACHE II score better predicts short-term outcomes in critically Ill patients: A retrospective cohort study. BMC Anesthesiol.

[15] Paal P, Gordon L, Strapazzon G (2016). Accidental hypothermia-an update: The content of this review is endorsed by the International Commission for Mountain Emergency Medicine (ICAR MEDCOM). Scand J Trauma Resusc Emerg Med.

[16] Minne L, Abu-Hanna A, de Jonge E (2008). Evaluation of SOFA-based models for predicting mortality in the ICU: A systematic review. Crit Care.

[17] Cohen IJ (2023). Prolonged hypothemic duration (PHD) causes delayed rewarming thrombocytopenia (DRT): A revolutionary new concept based on five novel observations. Am J Emerg Med.

[18] Siren J, Vaahersalo J, Skrifvars M, Pettilä V, Tiainen M, Tikkanen I, Lakkisto P (2016). Plasma heme oxygenase-1 in patients resuscitated from out-of-hospital cardiac arrest. Shock.

[19] Attuwaybi BO, Kozar RA, Moore-Olufemi SD, Sato N, Hassoun HT, Weisbrodt NW, Moore FA (2004). Heme oxygenase-1 induction by hemin protects against gut ischemia/reperfusion injury. J Surg Res.

